# Supplementary material for: The interactome of intact mitochondria by cross-linking mass spectrometry provides evidence for coexisting respiratory supercomplexes
Source: Mol Cell Proteomics. 2017 Dec 8;17(2):216–32. doi: 10.1074/mcp.RA117.000470 (PMC5795388; doi:10.1074/mcp.RA117.000470)
Supplement: Supplemental Data [file supp_RA117.000470_133922_0_supp_23978_fzffwf.zip › spectra_annotation/mito_spectra_annotation/110-1-4-1-14-1.pdf]

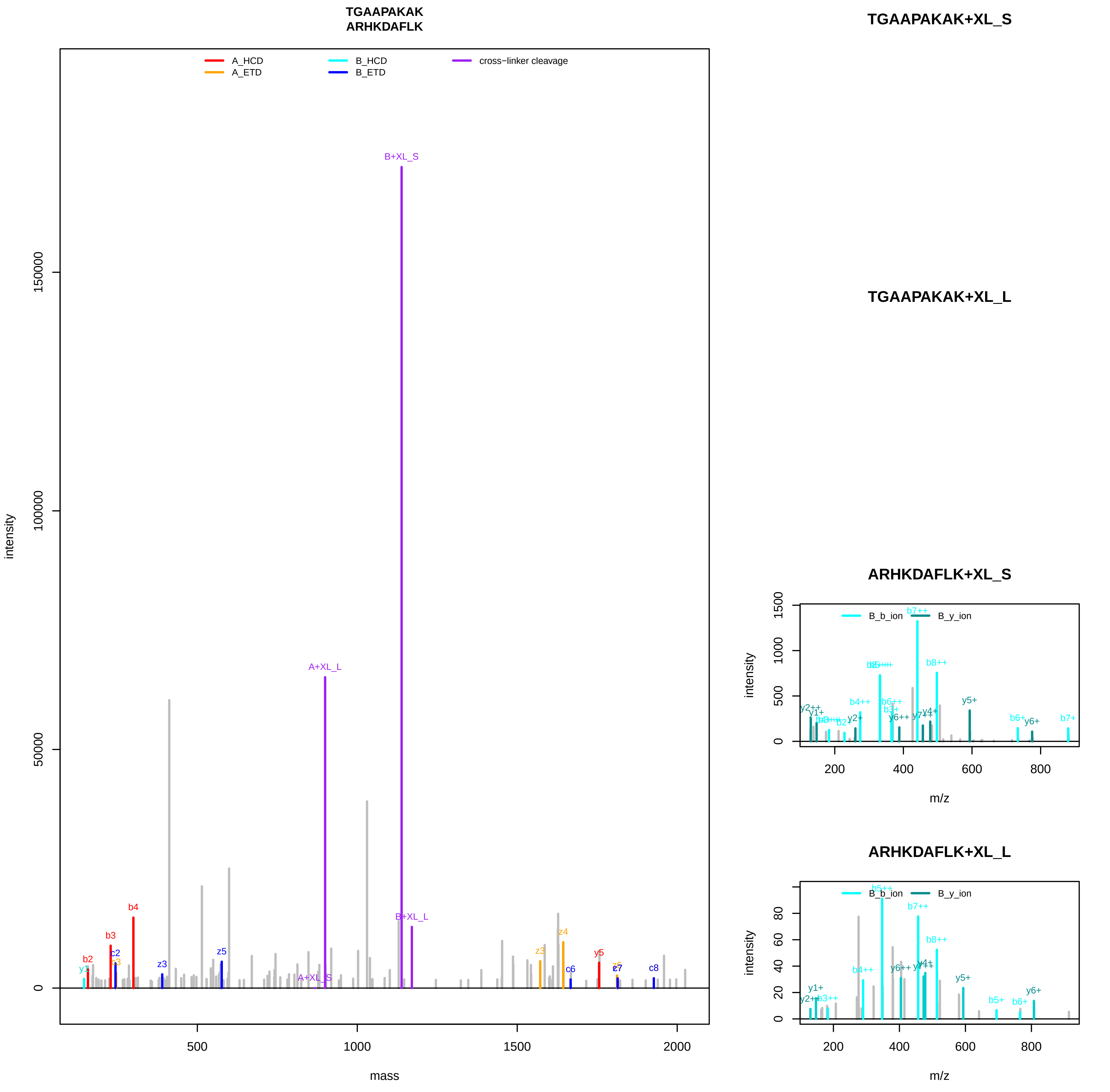
TGAAPAKAK
ARHKDAFLK
A_HCD
A_ETD
B_HCD
B_ETD
cross−linker cleavage
B+XL_S
A+XL_L
B+XL_L
A+XL_S
b2
b3
b4
y1
c2
z3
z5
z3
z4
y5
c6
c7
c8
intensity
mass
0
50000
100000
150000
500
1000
1500
2000
TGAAPAKAK+XL_S
TGAAPAKAK+XL_L
ARHKDAFLK+XL_S
ARHKDAFLK+XL_L
B_b_ion
B_y_ion
b7++
b8++
b4++
b6++
y5+
b6+
y6+
b7+
b5++
y1+
y6++
y4+
b5+
intensity
m/z
0
500
1000
1500
0
20
40
60
80
200
400
600
800
